# Whole brain radiotherapy for brain metastases in Nigeria: a systematic review

**DOI:** 10.3389/fpubh.2026.1868865

**Published:** 2026-06-15

**Authors:** Kehinde Alare, Nenkimun Dirting Bakwa, Izuchukwu Okechukwu Nwachukwu, Deborah Ngozi Conteh, Abdulrahmon Moradeyo, Mubarak Jolayemi Mustapha, Abdulbasit Muili, Samson Adedeji Afolabi, Marlin Mazima, Fan Chen

**Affiliations:** 1 Department of Medicine, Ladoke Akintola University of Technology, Ogbomoso, Nigeria; 2 Division of Neurosurgery, Department of Surgery, Jos University Teaching Hospital, Jos, Nigeria; 3 Division of Public Health, Sport and Wellbeing, University of Chester, Chester, United Kingdom; 4 Department of Surgery, University of Ilorin teaching Hospital, Ilorin, Kwara state, Nigeria; 5 Department of Neurosurgery, The Second Affiliated Hospital Of Air Force Medical University, Xi'an, Shaanxi, China; 6 Department of Medicine, Kabale University, Kabale Municipal, Uganda

**Keywords:** brain metastases, Nigeria, systematic review, WBRT (Whole brain Radiotherapy), whole brain radiotherapy

## Abstract

**Background:**

Whole brain radiotherapy (WBRT) remains the most commonly used treatment for brain metastases in Nigeria due to limited access to advanced radiotherapy techniques. However, evidence on its outcomes within the Nigerian context has not been systematically summarized.

**Methods:**

A PRISMA-guided systematic review was conducted to identify studies reporting the use of WBRT for brain metastases in Nigeria by systematic searching of PubMed/Medline, Google Scholar, Embase, and Ajol. Data were extracted on study characteristics, primary tumor profiles, treatment protocols, clinical outcomes, Karnofsky and WHO performance status, and toxicities.

**Results: Four:**

studies were included in this review, involving a total of 142 patients, with 128 females accounting for 90.1% of the sample size. The mean age was 47.3 ± 12.4 years. CT scan accounts for 70.6% of investigation modalities, MRI (23.5%), Both (5.9%). Mean KPS is 50, breast Cancer accounts for 82.4% of the source of metastases, 66.7% of primary tumors are in stage 3. Multiple metastatic lesions are seen in 85.9% of cases, while 76.9% of cases are supratentorial. Steroid (35%), surgery (9%), and chemotherapy (3%) are additional therapies received. Median survival period ranges between 3 to 18 months with a pooled median survival period of 3.6 months (95% CI: 2.8, 4.4), complete clinical response is seen in 53.8% of patients, with whole brain radiotherapy and improvements in WHO performance status following therapy.

**Conclusion:**

WBRT continues to provide symptomatic benefit for patients with brain metastases in Nigeria, but is limited by modest survival outcomes and gaps in outcome reporting. Improved access to advanced radiotherapy techniques and standardized reporting are needed to optimize care.

## 1 Introduction

### 1.1 Burden of brain metastases globally and in Nigeria

Brain metastases represent a critical oncologic challenge worldwide. They are the most common intracranial tumors in adults, occurring in approximately 20% of all patients with cancer, and their incidence continues to rise as systemic disease control improves and patient survival increases (1).

The primary cancers most frequently associated with brain metastases are lung cancer, followed by breast cancer and melanoma, with renal and colorectal cancers also contributing to the burden of intracranial metastatic disease (2). These metastases substantially contribute to morbidity, neurologic dysfunction, and short median survival measured in months absent effective intervention (1).

In Nigeria and other low-resource regions, comprehensive national epidemiologic data on brain metastases are limited. Although formal reporting systems such as the National Cancer Registry exist, population-level surveillance remains incomplete, impeding precise estimation of incidence and trends (3). However, available regional data and hospital-based case series indicate that brain metastases are not uncommon and frequently present at advanced stages, exacerbated by delays in diagnosis and limited access to neuroimaging (e.g., Magnetic resonance imaging) (4). A systematic review of radiotherapy and radiosurgery across Africa noted that metastatic tumors comprised a large proportion of brain tumor cases in Nigerian series, with breast cancer being a predominant primary source (5). While in Nigeria, a systematic review and pooled analysis of hospital-based records reported that brain metastases accounted for 8% of all intracranial neoplasms (6, 24).

### 1.2 Role of radiotherapy in brain metastases

Radiotherapy is a foundational modality in managing brain metastases, particularly in palliative settings. Historically, whole brain radiotherapy (WBRT) has served as the standard approach for patients with multiple intracranial lesions or when metastatic disease is widespread. WBRT delivers fractionated radiation to the entire brain, addressing both overt lesions and microscopic spread beyond visible metastases (7).

Clinical evidence indicates that WBRT can alleviate neurologic symptoms, improve disease control within the brain, and extend survival relative to supportive care alone for selected patients (8). The addition of WBRT to focal treatments like stereotactic radiosurgery (SRS) may improve local and distant intracranial control, although it has not consistently demonstrated a survival advantage in randomized studies (9). Furthermore, WBRT is associated with risks of neurocognitive decline and quality-of-life impairments, which have contributed to increasing use of SRS and other targeted approaches when resources permit (9).

Despite these concerns, WBRT remains a key option, especially when multiple metastases preclude focal techniques or when rapid symptomatic relief is required (10).

### 1.3 Rationale for continued use of WBRT in resource-limited settings

In many resource-limited settings, including Nigeria, WBRT continues to be widely utilized due to practical considerations in local oncology practice. High-precision radiotherapy modalities such as SRS require advanced equipment, specialized planning systems, and trained personnel, which are scarce in low- and middle-income countries (5). By contrast, WBRT can be delivered using more commonly available external beam radiotherapy infrastructure, making it a feasible and accessible option in hospitals with limited technological capacity (5).

The predominance of late-stage presentation with multiple brain lesions further supports the use of WBRT, as its broad field of radiation can target both identifiable and microscopic disease (5). Moreover, studies from Nigerian centers demonstrate that WBRT can improve patient-reported outcomes, including symptom burden and functional status, even when overall survival remains modest (11). In prospective data from a tertiary Nigerian cancer center, patients treated with WBRT reported significant improvements in physical and emotional functioning up to 6 months after therapy (11).

### 1.4 Knowledge gap and study objective

Despite the continued reliance on WBRT in Nigeria, consolidated evidence on its utilization, clinical outcomes, and context-specific relevance remains scarce. The current literature on WBRT in Nigeria consists largely of single-institution reports and small observational series, with limited synthesis of outcomes, treatment patterns, or comparative evaluations with alternative approaches (5). The scarcity of systematic data hinders evidence-based practice and policy development in neuro-oncology for low-resource environments.

This systematic review aims to address these gaps by identifying, appraising, and synthesizing existing studies concerning WBRT for brain metastases in Nigeria. The objectives are to:

1. Characterize patterns of WBRT use in clinical practice across Nigerian treatment centers.
2. Evaluate reported clinical outcomes and adverse effects, including symptom control, survival, and quality of life.
3. Highlight evidence gaps and areas necessitating further research to inform policy and optimize care delivery.

Through this review, we seek to provide a comprehensive evidence base that can support clinicians, researchers, and policymakers in improving neuro-oncologic care in resource-limited settings.

## 2 Methods

### 2.1 Study design

This systematic review was conducted based on the Preferred Reporting Items for Systematic Reviews and Meta-Analyses (PRISMA) guidelines (12). The research protocol was registered with open science framework doi: 10.17605/OSF.IO/AJQNE, https://osf.io/ajqne.

### 2.2 Search strategy

A systematic search was made on PubMed/Medline, Google Scholar, Scopus, Embase, Cochrane Library, and AJOL with themes on state and outcomes of whole brain radiotherapy for brain metastases in Nigeria from inception till September 2025. The search strings per database are reported in Table 1 below.

**Table 1:** Search Strings per databases.

Database	Search strings
PubMed/Medline	Primary: (“Radiotherapy, Whole-Brain”[MeSH] OR “Radiotherapy, Whole Brain”[Title/Abstract] OR “WBRT”[Title/Abstract]) AND (“Brain Neoplasms”[MeSH] OR “brain metastases”[Title/Abstract]) AND (“Nigeria”[MeSH] OR “Nigeria”[Title/Abstract]) Broader: (“radiotherapy”[Title/Abstract] OR “irradiation”[Title/Abstract]) AND (“brain metastases”[Title/Abstract] OR “cerebral metastases”[Title/Abstract]) AND (Nigeria OR Nigerian)
Google scholar	Primary: “whole brain radiotherapy” OR “whole-brain radiotherapy” OR WBRT “brain metastases” OR “cerebral metastases” Nigeria OR Nigerian Broader: radiotherapy “brain metastases” Nigeria
Embase	(‘whole brain radiotherapy'/exp OR ‘whole brain radiotherapy' OR ‘WBRT' OR ‘brain irradiation' OR ‘cranial radiotherapy') AND (‘brain metastasis'/exp OR ‘brain metastasis' OR ‘cerebral metastasis' OR ‘metastatic brain tumour') AND (‘nigeria'/exp OR ‘nigeria' OR ‘nigerian')
Scopus	(TITLE-ABS(“whole brain radiotherapy” OR “whole-brain radiotherapy” OR WBRT OR “brain irradiation” OR “cranial radiotherapy”)) AND (TITLE-ABS(“brain metastases” OR “cerebral metastases” OR “metastatic brain tumor”)) AND (TITLE-ABS(Nigeria OR Nigerian))
Web of science	TS=(“whole brain radiotherapy” OR “whole-brain radiotherapy” OR WBRT OR “brain irradiation” OR “cranial radiotherapy”) AND TS=(“brain metastases” OR “cerebral metastases” OR “metastatic brain tumor”) AND TS=(Nigeria OR Nigerian)
Cochrane library	(“whole brain radiotherapy” OR “whole-brain radiotherapy” OR WBRT OR “brain irradiation”) AND (“brain metastases” OR “cerebral metastases”) AND (Nigeria OR Nigerian)
AJOL	“whole brain radiotherapy” OR “whole-brain radiotherapy” OR WBRT OR “brain irradiation” AND “brain metastases” OR “cerebral metastases” AND Nigeria OR Nigerian
ClinicalTrials.gov	whole brain radiotherapy AND brain metastases AND Nigeria

AJOL, African Journals Online.

### 2.3 Eligibility criteria

Following the PICO framework, the eligibility criteria are as follows:

Population: Patients with metastatic brain lesion in managed in Nigeria.

Inclusion criteria:

- Nigerian patients.
- Patients referred for management in Nigeria.

Exclusion Criteria:

- Patients who received management outside Nigeria.
- Animal subjects or models.
- Patients with spinal metastatic lesions.
- Patients with primary brain tumors or CNS lymphoma.

Intervention: Whole brain radiotherapy.

Inclusion criteria:

- Radiation therapy for brain lesions as the main therapy.
- Radiotherapy as an adjuvant to surgery or chemotherapy.

Exclusion Criteria:

- Craniospinal Irradiation.

Comparative: Not applicable.

Outcomes: Median survival period, WHO performance status, safety/ side effects.

Other eligibility Criteria:

- Types of studies are randomized controlled trials, cohort studies, case-control studies, case series, and case reports.
- The language of publication is English.

### 2.4 Study selection

**Figure 1:**
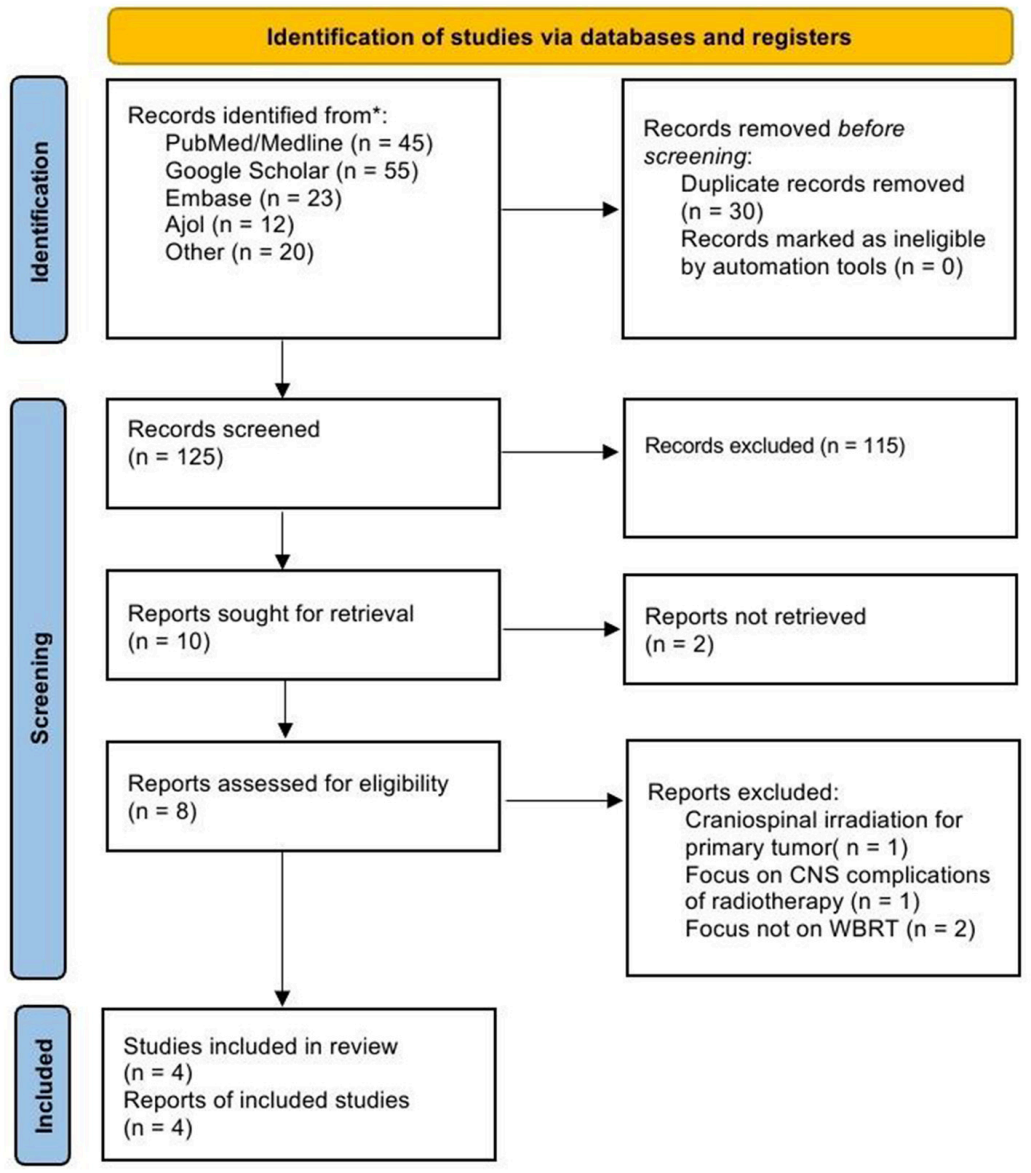
PRISMA flow diagram.

Following duplicates exclusion with Mendeley (Elsevier, London, UK), article screening was done in two stages: the title and abstract screening, and full texts screening. Groups of a pair of reviewers independently screened a set of articles using the Rayyan software. The screening process is blinded and unblinded by end of the screening and a third reviewer joined to aid conflict resolution. The PRISMA flow diagram of the screening process is shown in Figure 1.

### 2.5 Data extraction

Two authors independently extracted data from included studies. The extracted data areAuthor, year, study type, sample size, patients' characteristics (Age, gender, comorbidities), tumor profiles (primary tumor site, and stage), about radiotherapy (dose, fraction) predictors of response (pre therapy KPS and WHO performance status), efficacy outcomes (Percentage of response, and responder rate, post therapy WHO performance status, median survival period), and safety outcomes (Quality of life assessment, and adverse effects). The data were input into a central excel sheetand exported for feasible statistical analysis.

### 2.6 Quality assessment

The strength of the chosen studies was evaluated using the Newcastle-Ottawa Scale (NOS). High-quality studies with a score of more than seven were included in the final analysis.

### 2.7 Data synthesis

Narrative synthesis of outcomes of whole brain radiotherapy for the management of brain metastases in Nigeria was done, while we did a limited quantitative summary of patients' demographics.

### 2.8 Data analysis

When feasible, descriptive statistics were used to evaluate continuous variables, which were then given as the median (range). The corresponding forest plot showed the pooled point estimate for our main endpoints was generated using STATA (version 14.0). Fekete et al. (23) previously detailed the process used to create these visualizations. In short, a random effects model was chosen, and each research input variable was combined into an overall point estimate using the inverse-variance weighting method.

## 3 Results

### 3.1 Characteristics of included studies

Initial search generated 155 articles, of which 4 articles with a total of 142 patients were included in this systematic review. These Studies were published between 2016 and 2023 and recruited patients between 2005 and 2020. Three of the included studies were done in southwestern Nigeria (11, 14, 15), and two are retrospective (13, 14), while two are prospective in nature (11, 15). Table 2 shows the full characteristics of the included studies.

**Table 2:** Characteristics of included studies.

Reference	Year	Study period	Study design	State	Geopolitical xone	Focus	Subject group	Sample size	Male/ female ratio	Mean age	Investigation	Important findings	NOS total score
Olaseinde et al., (13)	2016	2006–2015	Retrospective	Kaduna	North West	Demographics	Mixed	30	5/25	43.5	NR	Breast Cancer is the most common tumor of origin in patients receiving Whole brain radiotherapy for Brain Metastases with the mean age of presentation at 43.5 years. Majority of Patients are females.30Gy in 10 # was the most common regimen received by Patients and the median survival for patients is 3 months	7
Ibrahim et al., (14)	2019	2005–2009	Retrospective	Oyo	South West	Therapeutic response	Adults	52	0/52	44.7 ± 12.5	CT scan and MRI	WBRT is an effective treatment modality for patients with brain metastasis in their resource-poor environment. Patients' performance status declines with advanced aging. The studies recommend supportive care alone without WBRT in older adults patients especially those older than 70 years	8
Adegboyega et al., (11)	2023	2020	Prospective	Lagos	South West	Patient's reported outcome	Adults	52	9/53	53 ± 12.3	NR	WBRT is an effective palliative intervention in patients with BM, resulting in improved QoL. More than 50% of patients that survived~3 months reported alleviation of pain, and 38% of patients that survived for~6 months reported a significantly improved functioning	9
Ayandapo et al., (15)	2019	2013–2016	Prospective	Oyo	South West	Clinical outcomes	Adults	8	0/8	41	CT scan and MRI	Whole brain radiotherapy prolonged survival among post surgical metastasectomy patients by 18 months. Prolonged survival is synergistic, as surgical intervention also prolonged survival. Patients who had surgery then whole brain radiotherapy had longer survival compared to those who had whole brain radiotherapy alone	8

NR, not reported; NOS, newcastle-ottawa scale.

### 3.2 Patient and tumor characteristics

This study included a total of 142 patients with 14 males (9.9%) and 128 females (90.1%), the mean age of included patients being 47.3 ± 12.4 years. Neuroimaging investigations, including brain CT scan and MRI are the main investigations carried out by patients as reported in Table 3.

**Table 3:** Baseline characteristics and investigation modalities.

Characteristic	Value
Total sample size	142
Total cases	142
Mean Age ± SD (Years)	47.3 ± 12.4
Gender distribution	
Male	14 (9.9%)
Female	128 (90.1%)
Subject group	
Adult	3 (75.0%)
Mixed	1 (25.0%)
Frequency of investigation modalities	
X-ray, *n* (%)	0 (0.0%)
CT Scan, *n* (%)	48 (70.6%)
MRI, *n* (%)	16 (23.5%)
CT + MRI, *n* (%)	4 (5.9%)
Bone Scan, *n* (%)	0 (0.0%)
Abdominal CT Scan, *n* (%)	0 (0.0%)

CT, computed tomographic; MRI, magnetic resonance imaging; SD: standard deviation.

Breast Cancer accounts for 82.4% of the primary sources of metastases, most primary tumors are found to be in stages 3 and 2, with a mean baseline Karnofsky Performance Scale of 50. The number of lesions were reported in 45% of included patients, of which 85.9% have multiple metastatic lesions, while 14.1% have single lesions. In 40% of cases in which the site of lesions was reported, lesions are supratentorial and unilateral. The size of lesions was reported in 12 patients, of which 58.3% are greater than 4cm, as shown in Table 4.

**Table 4:** Lesion characteristics and tumor profile.

Characteristic	Value
Number of Lesions–Single	9 (14.1%)
Number of Lesions–Multiple	55 (85.9%)
Site of Lesions–Supratentorial	10 (40.0%)
Site of Lesions–Infratentorial	2 (8.0%)
Site of Lesions–Both	1 (4.0%)
Site of Lesions–Unilateral	10 (40.0%)
Site of Lesions–Bilateral	2 (8.0%)
Size of Lesions–Diameter > 4 cm	5 (41.7%)
Size of Lesions–Diameter < 4 cm	7 (58.3%)
Mean KPS	50.0
Primary tumor site	
Breast	117 (82.4%)
Prostate	1 (0.7%)
Renal	1 (0.7%)
Lung	8 (5.6%)
Head and Neck	4 (2.8%)
Colon	2 (1.4%)
Musculoskeletal	1 (0.7%)
Lymphoma	2 (1.4%)
Other^*^	6 (4.2%)
Frequency of stages of tumor	
Stage 1	0 (0.0%)
Stage 2	4 (33.3%)
Stage 3	8 (66.7%)
Stage 4	0 (0.0%)

Other^*^: Pancreatic, bone, endometrial, and paraanals.

KPS, Karnofsky Performance Scale.

### 3.3 Treatment characteristics

External beam radiotherapy was the most common form of whole brain radiotherapy techniques. Surgeries, chemotherapy, and steroids are additional therapies received by patients as reported in Table 5.

**Table 5:** Treatment characteristics, response, and outcomes.

Characteristic	Value
Frequency of treatment modes	
Radiotherapy	142 (100%)
Surgery	12 (8.5%)
Chemotherapy	4 (2.8%)
Steroids	50 (35.2%)
Cumulative mean dose of radiation	30.8 Gy
Cumulative mean fraction of radiation	9.7
Mean treatment duration	21.0 days
Therapeutic response	
Complete response	53.8%
Partial response	32.7%
Lost to follow-up	13.5%
WHO performance status before therapy	
Status (0–2)	46.2%
Status (3–4)	53.8%
WHO performance status after therapy	
Status (0–2)	86.5%
Status (3–4)	0%
Lost to Follow-up	13.5%
Median survival duration	3.6 months (95% CI: 2.8–4.4)

The mean dose of radiotherapy is 30.8Gy which ranges from 27.6 to 34 Gy. The fractionated pattern showed a mean fraction of 9.7, mean duration of treatment is 21 days, as shown in Table 5.

### 3.4 Clinical outcomes

**Figure 2:**
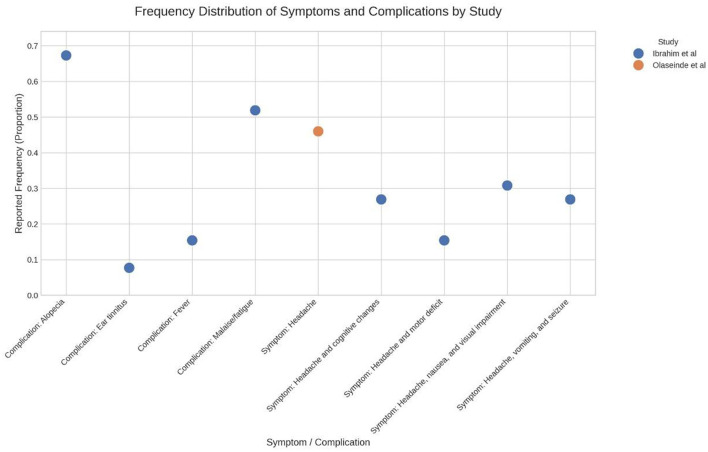
Schematic representation of frequency of distributions of symptoms and complications.

Complete clinical Response with symptoms are seen in 53.8% of cases; the majority of patients had a WHO performance status score between 3 to 4 prior to WBRT, while most patients had a score between 0 to 2 after WBRT (14). The median survival duration ranges between 3 to 18 month with a pooled median survival period of 3.6 months (95% CI: 2.8–4.4 months, I^2^ 70.74%)are seen in patients who had WBRT, as shown in Figure 2. Patients reported outcome: approximately 38% of patients who lived for up to 180 days reported improved functioning and reduced symptoms (11). At 180 days after WBRT, there was a significant improvement in both physical and emotional functioning (*p* < 0.001 and *p* = 0.031, respectively) compared to baseline, and improved QoL was reported in over 50% of patients (11).

### 3.5 Treatment-related toxicities

**Figure 3:**
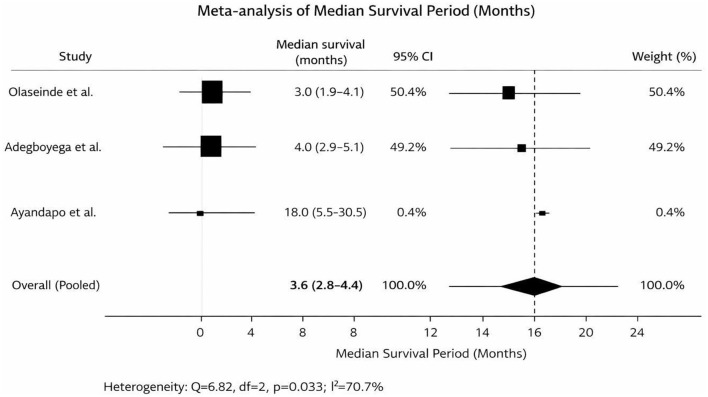
Forest plot of median survival period in months.

Common adverse effects reported are alopecia, malaise, fever, and tinnitus, as shown in Figure 3, no severe neurocognitive toxicities were reported in the included studies; however, formal cognitive assessments were largely absent.

### 3.6 Quality assessment

The NOS of included studies are shown in Supplemental Table.

## 4 Discussion

This review summarizes the clinical landscape in Nigeria for the use of WBRT for brain metastases. Our findings shed light on a challenging clinical reality in which the presentation of advanced disease, demographic differences, and significant resource constraints all combine to influence patient outcomes.

The demographic profile of the patients in this review is significantly different from global trends. While lung cancer is the most prevalent cause of brain metastases in many Western and Asian populations (16), breast cancer prevailed in Nigeria, accounting for 82.4% of cases. This is consistent with regional findings from Ibrahim and Yaroko (14), who found that the high prevalence of advanced breast cancer in Nigeria frequently results in neurological problems. The average age of 47.3 years is also significantly lower than the 55–60 year range commonly reported in high-income countries (HICs), indicating that metastatic illness is affecting a younger, more productive population in West Africa (17). Clinically, the severity of the disease at presentation is significant. The mean Karnofsky Performance Status (KPS) of 50.0 and the presence of numerous metastatic lesions in 85.9% of cases suggest that most patients seek medical attention only after considerable neurological deterioration. This is in stark contrast to worldwide standards, which progressively reserve WBRT for patients with more than 4–10 lesions, while those with fewer lesions are treated with Stereotactic Radiosurgery (SRS) (18). In Nigeria, due to a lack of SRS infrastructure, WBRT is the default standard. Despite the late presentation, WBRT had a therapeutic benefit, with 53.8% of patients attaining a complete clinical response and improved performance status.

Our findings confirm that WBRT remains an essential palliative therapy in the Nigerian oncological toolkit. The high incidence of clinical response shows that radiotherapy is beneficial in relieving uncomfortable symptoms such as intracranial pressure (ICP), seizures, and focused impairments, even if treatment does not appreciably prolong life (19). As a result, improving the “time to radiation,” the time between diagnosis and treatment initiation, is crucial. The overall data point to a need for more aggressive use of adjunctive therapies; the low utilization of steroids (35%) and surgery (9%) suggests that many patients may not be receiving the full range of available supporting services. Integrating palliative care specialists early in the treatment pathway may enhance symptom control and increase quality of life throughout the 180-day survival window found in some patients (20).

The strong reliance on WBRT in Nigeria is the direct result of structural resource constraints. CT scans are the most common diagnostic modality (70.6%), while MRI is employed in just 23.5% of patients. In high-resource settings, MRI is the gold standard because its superior sensitivity frequently shows tiny metastases overlooked by CT, potentially altering the treatment approach (21). Furthermore, most radiation centers in Nigeria have traditionally used Cobalt-60 devices and 2D planning (22). This lacks the precision of 3D-conformal or Volumetric Modulated Arc Therapy (VMAT), which are available in HICs and enable approaches such as hippocampal-sparing WBRT to lessen neurocognitive side effects.

Due to the paucity of SRS-capable linear accelerators and the advanced stage of disease at presentation, the “dwindling” function of WBRT seen in international literature driven by worries regarding cognitive decline, is not yet a realistic reality in Nigeria. Furthermore, the gaps in outcome reporting found in this review are largely caused by the financial strain placed on patients in a primarily out-of-pocket payment (OOPs) system, which frequently prevents the completion of treatment or follow-up.

## 5 Limitations

There were several limitations to the study that may have influenced the outcomes. Firstly, the methodologies across the included studies varied considerably, introducing a high level of heterogeneity. Furthermore, the total number of patients included in the systematic review was 142. This may be attributable to the paucity of data from certain geopolitical regions of the country, as three-quarters of the studies were conducted in centers located in the southwestern region.

Although this study represents the first review examining Whole Brain Radiotherapy in Nigeria, the relatively small sample size should be considered, as this may limit the generalisability of the findings. Nonetheless, it highlights important gaps in the existing literature and opens avenues for further research. Finally, the majority of the study population was female. This finding is expected, given that breast cancer remains the most common cause of brain metastases within the region. However, this gender imbalance further limits the extent to which the findings can be fully generalized.

Overall, this study represents a step in the right direction, and the identified gaps and limitations provide a foundation for future research.

## 6 Future directions

The findings of this systematic review underscore the persistent reliance on whole brain radiotherapy (WBRT) as a palliative modality for brain metastases in Nigeria, while highlighting substantial gaps in infrastructure, outcome reporting, and access to advanced therapies. To optimize patient care and align with evolving global standards, several key areas warrant prioritization in future research and policy initiatives:

Conduct Multicenter Prospective Studies: Current evidence is limited to small, predominantly retrospective series from southwestern Nigeria. Large-scale, multicenter prospective cohort studies or registries encompassing all geopolitical zones are essential to capture national trends, refine prognostic models (e.g., incorporating local factors like late presentation and primary tumor profiles), and evaluate long-term outcomes beyond median survival.

Expand Access to Advanced Radiotherapy Techniques: The predominance of WBRT reflects resource constraints, with limited availability of stereotactic radiosurgery (SRS) or stereotactic radiotherapy. Investments in linear accelerator-based SRS programs, supported by international collaborations (e.g., IAEA Rays of Hope) and public-private partnerships, could enable focal treatments for oligometastatic disease, potentially improving intracranial control while reducing neurocognitive risks compared to WBRT.

Incorporate Routine Neurocognitive and Quality-of-Life Assessments: Formal neurocognitive testing was largely absent in included studies, despite known risks of WBRT-related decline. Future protocols should integrate validated tools (e.g., Mini-Mental State Examination, Hopkins Verbal Learning Test) and patient-reported outcomes to quantify cognitive sequelae and guide neuroprotective strategies, such as memantine prophylaxis or hippocampal avoidance where feasible.

Strengthen Multimodal and Supportive Care Integration: Low utilization of adjunctive therapies (e.g., steroids, surgery) suggests opportunities for standardized protocols combining WBRT with systemic agents (e.g., targeted therapies for breast cancer metastases) and early palliative care to enhance symptom control and functional status.

Address Infrastructure and Equity Gaps: Decentralization of radiotherapy services, workforce training (e.g., medical physicists, radiation oncologists), and affordable neuroimaging (e.g., wider MRI access) are critical to reduce delays in diagnosis and treatment initiation. National cancer control plans should prioritize equitable distribution of facilities and explore cost-effective innovations tailored to low-resource settings.

By pursuing these directions, Nigeria can build a more robust evidence base and transition toward personalized, less toxic management of brain metastases, ultimately improving survival and quality of life for affected patients.

## 7 Conclusion

Whole Brain Radiotherapy plays a significant role in the regression and control of metastatic brain lesions, as demonstrated by the findings of this study. Although its utilization in Nigeria remains relatively nascent, there appears to be a positive trend regarding its effects on symptom management, especially as palliative therapy. Despite these benefits, access to Whole Brain Radiotherapy remains limited, with services neither widely available nor equitably distributed across the region.

Consequently, greater attention should be directed toward addressing the factors influencing the accessibility, affordability, and availability of Whole Brain Radiotherapy services. Improving these determinants may facilitate earlier access to care, particularly given that late health-seeking behavior was identified as a contributing factor to the significant cognitive decline observed among patients with metastatic brain tumors.

Furthermore, the expansion and wider geographical distribution of Whole Brain Radiotherapy facilities would ensure improved patient access and maximize the potential benefits of this treatment modality. Finally, there remains a substantial paucity of evidence within the region, underscoring the need for further research to better elucidate the clinical outcomes, long-term benefits, and broader impact of Whole Brain Radiotherapy in this setting.

## Data Availability

The original contributions presented in the study are included in the article/supplementary material, further inquiries can be directed to the corresponding author.
